# Transcutaneous vagus nerve stimulation improves Long COVID symptoms in a female cohort: a pilot study

**DOI:** 10.3389/fneur.2024.1393371

**Published:** 2024-05-02

**Authors:** Zhong Sheng Zheng, Ninette Simonian, Jing Wang, Emily R. Rosario

**Affiliations:** ^1^Research Institute, Casa Colina Hospital and Centers for Healthcare, Pomona, CA, United States; ^2^Institute of Advanced Consciousness Studies, Santa Monica, CA, United States

**Keywords:** Long COVID, transcutaneous vagus nerve stimulation (t-VNS), female health, mood and cognition, sleep, fatigue, neuromodulation

## Abstract

**Background:**

Long COVID, also known as Post-COVID-19 syndrome, is characterized by multisystemic symptoms that persists for weeks to years beyond acute infection. It disproportionately affects women and those with pre-existing anxiety/depression, conditions more prevalent in females. The vagus nerve, with its extensive innervation and regulation of critical bodily functions, has become a focal point for therapeutic interventions. Transcutaneous vagus nerve stimulation (t-VNS) has emerged as a promising non-invasive treatment for COVID-19 conditions.

**Methods:**

This pilot study assessed the efficacy of t-VNS in 24 female Long COVID patients (45.8 ± 11.7 years old; 20.2 ± 7.1 months since infection), who underwent a 10-day t-VNS intervention at home (30 min/session, twice a day). Cognition was considered the primary outcome, with anxiety, depression, sleep, fatigue, and smell as secondary outcomes. Outcomes were measured at baseline, post-intervention, and 1-month follow-up.

**Results:**

Significant improvements were observed in various cognitive functions, anxiety, depression, and sleep at post-intervention, with benefits remaining or progressing at 1-month follow-up. Improvements in fatigue were delayed, reaching statistical significance at 1-month follow-up compared to baseline. No significant changes were noted in olfactory performance.

**Conclusion:**

This pilot study provides preliminary evidence supporting the potential of t-VNS as a therapeutic intervention for female Long COVID patients. The encouraging results justify further rigorous investigation through larger, randomized controlled trials to confirm the efficacy of t-VNS, assess its generalizability to male cohorts, and explore biological markers to inform personalized treatment approaches. Our findings support the allocation of resources to conduct such trials and advance the understanding of t-VNS as a potential treatment for Long COVID.

## 1 Introduction

Post-COVID-19 syndrome, commonly known as Long COVID, is characterized by a constellation of symptoms that persist for weeks to years after initial COVID-19 infection. According to the World Health Organization, Long COVID is defined by the presence of new or continuing symptoms, usually 3 months from acute infection, lasting at least 2 months without an alternative diagnosis (1). Estimates of Long COVID prevalence vary widely, partly due to the variability in definition and samples studied. Prevalence estimates range from 7.5% to 41% in non-hospitalized adults and 37.6% in hospitalized adults (2). With its recent emergence, insights into Long COVID’s underlying mechanisms are limited, hindering the advancement of targeted treatments.

While the prevalence of COVID infection is similar for both sexes, accumulating evidence suggests that females are disproportionately affected by Long COVID (3) and are more likely than males to suffer from hyposmia/anosmia and neurological symptoms (4–7). Pre-existing conditions such as anxiety or depression, which are more prevalent in women, further heighten the risk of developing Long COVID (7). Although the exact mechanisms of Long COVID remain unclear, these observations have led to the hypothesis that Long COVID could be, in part, related to autoimmunity and persistent inflammation (8), which might explain the higher incidence of this syndrome in women, as immune response for both genetic and hormonal factors is stronger in women (9, 10).

Cognitive impairment (e.g., attention or memory issues), commonly reported as “brain fog,” is highly prevalent in Long COVID, affecting approximately 80% of long haulers (11). Neuroimaging studies have detected damages in various limbic and associative brain regions following COVID-19 infection, likely indicative of neuroinflammation and possible neurodegeneration (12, 13). Notably, chronic loss of smell correlates with cognitive decline and is an early predictor of Alzheimer’s disease (14). Female sex, as well as early presence of olfactory and neurological symptoms, are well-known risk factors for dementia and Alzheimer’s disease (15, 16). Thus, the intersection of female sex and post-COVID-19 could potentially exacerbate this vulnerability. Prompt and effective management of Long COVID symptoms, with an emphasis on cognitive deficits, is essential to potentially reduce the risk of long-term neurological sequelae.

The vagus nerve, a key component of the parasympathetic nervous system, plays a vital role in regulating various bodily functions, including heart rate, respiration, digestion, mood, immune response, and more (17). Vagus nerve stimulation (VNS), or vagal neuromodulation, has been utilized as a therapeutic intervention for a wide range of neurological and psychiatric disorders, expanding the scope of VNS beyond its initial indications for epilepsy and treatment-resistant depression (18–22). In the context of COVID-19, non-invasive VNS, such as transcutaneous auricular vagus nerve stimulation (t-VNS), has emerged as a potential adjunct therapy, with studies exploring its anti-inflammatory effects and its ability to reduce the burden of COVID-19 (23, 24). Considering the immense overlap between vagal functions and post-COVID-19 symptoms, the use of t-VNS could potentially mitigate the multi-organ dysfunction seen in long haulers. Indeed, a recent pilot study demonstrated that t-VNS could reverse many of the symptoms of Long COVID (25); however, the cognitive impacts were not thoroughly evaluated. Moreover, emerging research has illuminated sex-dependent autonomic responses to t-VNS (26), suggesting that females may experience different therapeutic effects compared to their male counterparts. This differential response is critical to consider, particularly in the context of Long COVID, where women are disproportionately affected. Such insights underscore the necessity of tailoring t-VNS protocols to optimize therapeutic outcomes across sexes. Due to limited resources and the pilot nature of this study, we focused on an exclusively female cohort to control for heterogeneity and maintain statistical power.

Therefore, the current prospective pilot study aims to investigate the efficacy of t-VNS in treating female patients with Long COVID, with an emphasis on cognitive impairments—a significant and debilitating aspect of post-COVID-19 symptomology. Secondary outcomes, including anxiety, depression, sleep, fatigue, and olfactory function, will also be assessed. By focusing on this particularly vulnerable group, our research seeks to not only alleviate the immediate burden of Long COVID but also to preemptively address the risk of subsequent neurodegenerative sequelae. Given the pressing need for effective interventions amidst Long COVID’s mounting public health burden, research focused on vulnerable patients who stand to benefit most is of critical importance.

## 2 Materials and methods

### 2.1 Participants

**Figure 1:**
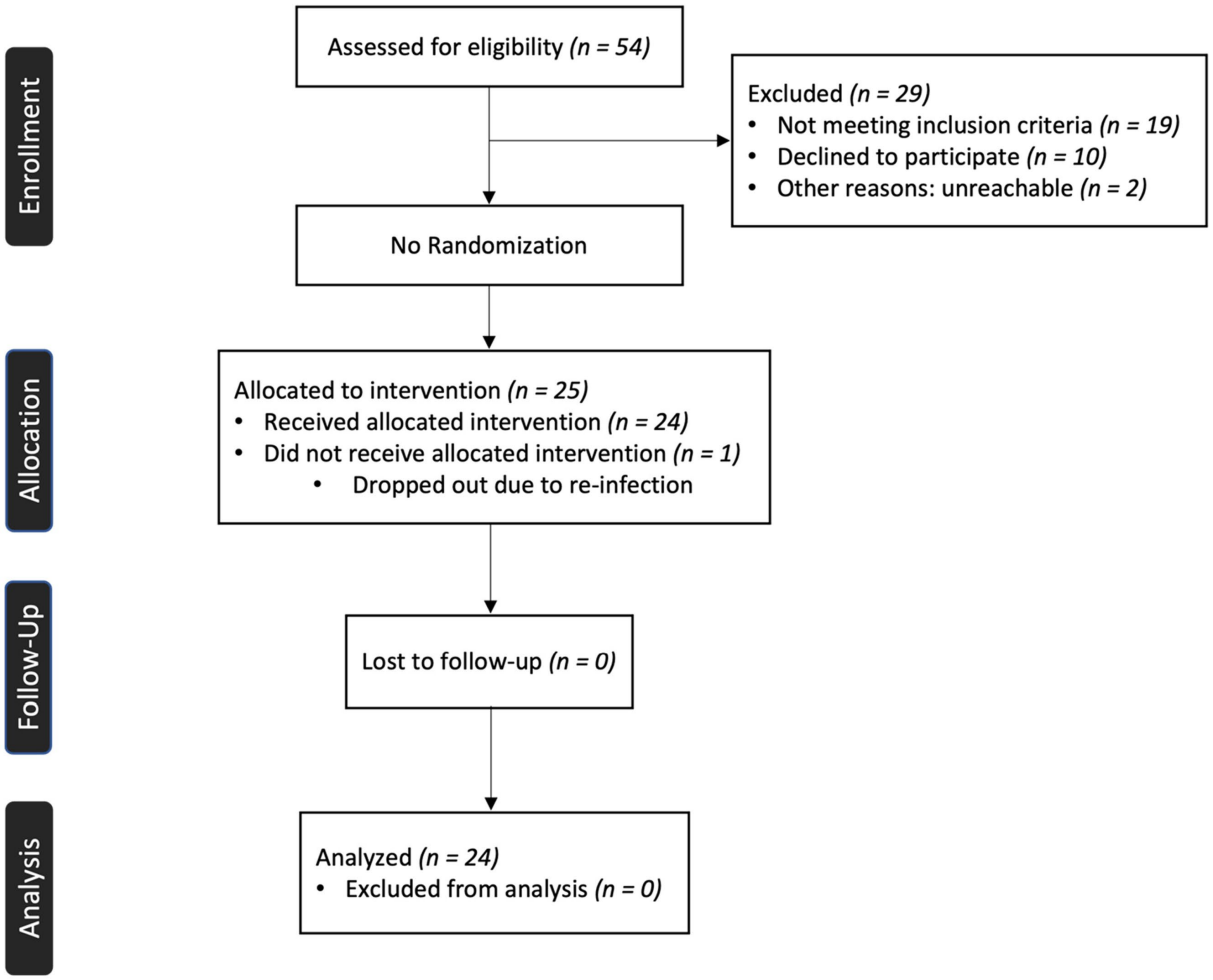
CONSORT flow diagram for a single-group pilot study.

This study included 24 female patients (45.8 ± 11.7 years of age) with persistent Long COVID symptoms, with an average length of 20.2 ± 7.1 months since COVID-19 infection. The inclusion criteria for participants were: female sex, 18 years or older, and experiencing persistent (more than 3 months after infection) cognitive impairment (“brain fog”), such as attention or memory deficits. Exclusion criteria included contraindications for vagal neuromodulation or magnetic resonance imaging, pregnancy, Long COVID without cognitive impairment, and a history of neurological conditions. Among the participants, 16.7% had pre-existing asthma, and 37.5% had pre-existing anxiety or depression. These conditions have been identified as risk factors for developing Long COVID (3, 27). Medication information is included in Supplementary Figure S1 and Supplementary Table S1. Data were collected between March 2022 and January 2023. The patient enrollment process is delineated in the CONSORT flow chart provided as Figure 1. This study was approved by the Institutional Review Board at Casa Colina Hospital and Centers for Healthcare. All participants provided written informed consent prior to participation (Registered on clinicaltrials.gov as NCT05225220).

### 2.2 Study procedure

Participants underwent 10 consecutive days of auricular vagal neuromodulation therapy (AVNT) at the comfort of their home, twice a day, once in the morning and once in the evening, for 30 min per session (60 min/day). Treatment was delivered with the Parasym AVNT system (Parasym Health, London, United Kingdom), which was designed specifically for use in this patient population. The electrode was applied to the left tragus of the ear, stimulating the auricular branch of the vagus nerve, with micro-pulses of current a proprietary waveform with a pulse width of 250 μs and a pulse frequency of 25 Hz. The current intensity used was personalized by the participants based on their own sensitivity threshold, setting the correspondent level on an interval scale at each treatment session by slowly increasing the current intensity until a constant tingling sensation was felt. The mean output current intensity across treatments was 13.64 mA.

### 2.3 Outcome measures

Behavioral assessments were carried out at three timepoints: pre-intervention, post-intervention, and 1-month follow-up. Six participants were from out-of-state and had their 1-month follow-up assessments completed remotely.

#### 2.3.1 Primary outcome measures

We used the NIH Cognitive toolbox to assess fluid cognition and included the following assessments: attention (Flanker Inhibitory Control and Attention Test [Flanker]), executive function (Dimensional Change Card Sort Test [DCCS]), episodic memory (Picture Sequencing Memory Test [PSM]), working memory (List Sorting Working Memory [LSWM]), and processing speed (Pattern Comparison Processing Speed [PCPS]). For the 1-month follow-up cognitive assessments, only LSWM and PSM were able to be administered remotely.

#### 2.3.2 Secondary outcome measures

We additionally explored secondary outcomes related to anxiety (BURNS Anxiety Inventory [range 0–56]), depression (Becks Depression Inventory-II [range 0–63]), fatigue (Fatigue Severity Scale [range 9–63]), sleep (PROMIS Sleep Disturbance—Short Form [range 8–40]), and smell (Sniffin’ Sticks Test—12 items [range 0–12]).

### 2.4 Data analysis

The data were analyzed in JASP to first determine normality, followed by parametric or nonparametric tests as appropriate. Parametric repeated-measures ANOVA (3 timepoints) was carried out for anxiety, depression, and fatigue scores, whereas non-parametric Friedman’s test was used for cognition, sleep, and smell. Upon finding significance, we ran parametric (T-test) or nonparametric (Wilcoxon’s rank sum) pairwise comparisons with Bonferroni correction between the three timepoints on all the outcome measures. Cohen’s d was used to calculate the effect size for parametric tests and rank biserial correlation (r) for nonparametric tests. For analyzing changes in cognitive scores, the uncorrected standard score was used. Due to limitations in remote testing for six participants, only 18 participants completed all cognitive and smell assessments at follow-up.

In our dataset, a few participants submitted incomplete questionnaire responses. To address the resultant missing data, we employed data imputation using the k-nearest neighbors (kNN) function in RStudio (28). A k-value of 5 was used to conduct the imputations (29). This procedure led to the imputation of 9 values across various domains: anxiety (3), sleep (3), depression (2), and fatigue (1). To assess the k-value used for our kNN imputation, we evaluated the accuracy using the Normalized Root Mean Square Error (NRMSE) by comparing imputed values against a subset of the dataset where actual values were known but temporarily treated as missing. Additionally, we conducted an analysis excluding participants with missing data, and the results were consistent with the results obtained using imputed data.

## 3 Results

Participants were tested at three time points: pre-intervention (baseline), post-intervention, and 1-month follow-up after 10 days of vagal neuromodulation. The primary outcome was cognition (attention, executive function, working memory, episodic memory, and processing speed). Secondary outcomes included anxiety, depression, sleep disturbance, and fatigue. Bonferroni-adjusted *p*-values are reported for *post hoc* tests.

### 3.1 Baseline clinical symptoms

**Figure 2:**
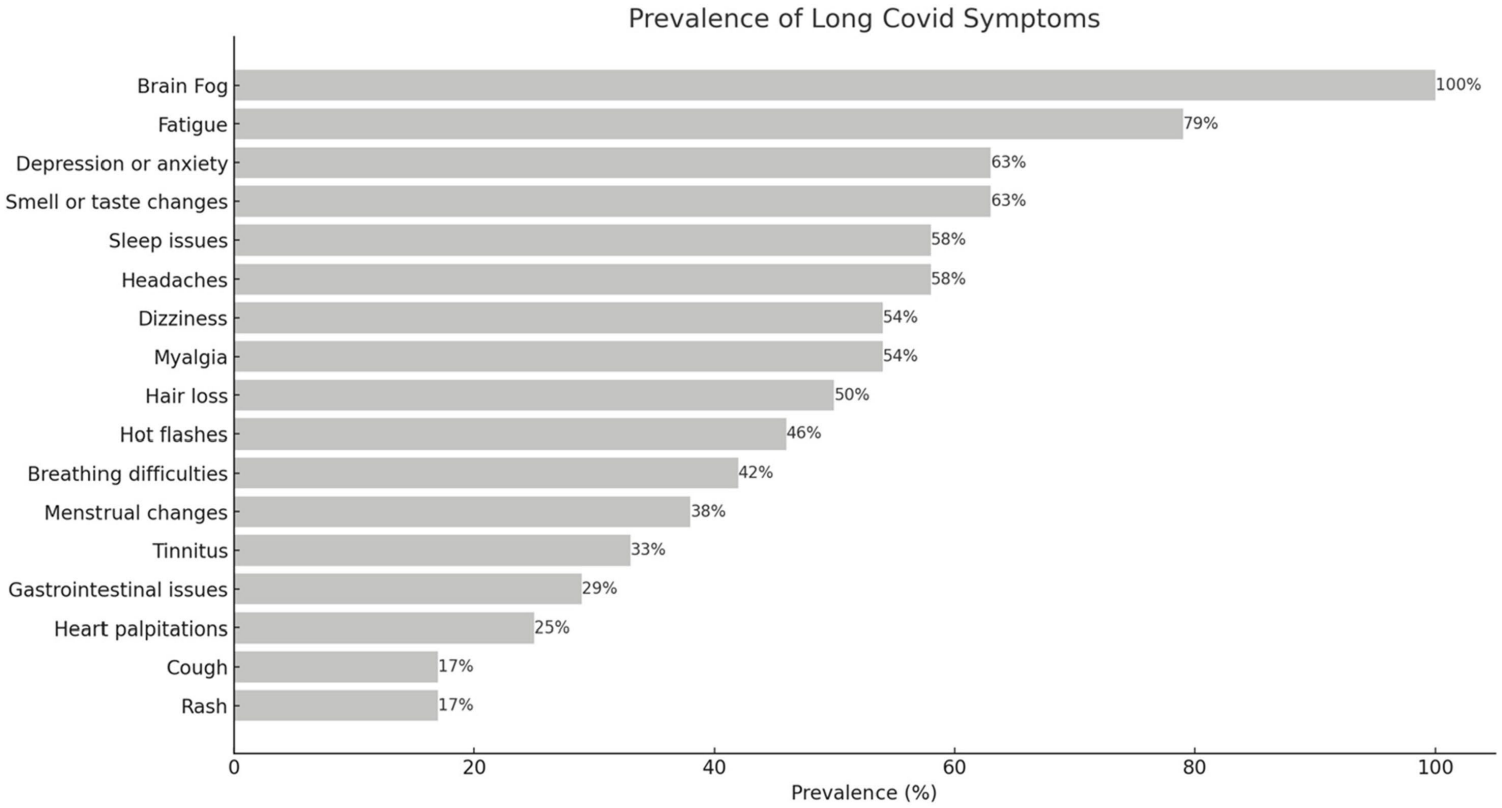
Prevalence of Long COVID symptoms at baseline. Brain fog (cognitive impairment) was an inclusion criterion; thus it is reported in all participants.

Prevalence of Long COVID symptoms at baseline are shown in Figure 2. The participants exhibited diverse symptoms across multiple systems. Following brain fog/cognitive impairment, which was an enrollment criterion, fatigue was the most common symptom, experienced by 79% of participants. Depression or anxiety and changes in smell or taste trailed behind (63%), then followed by headaches and sleep issues (58%). Please refer to Figure 2 for the extensive list of symptoms.

### 3.2 Primary outcome

**Figure 3:**
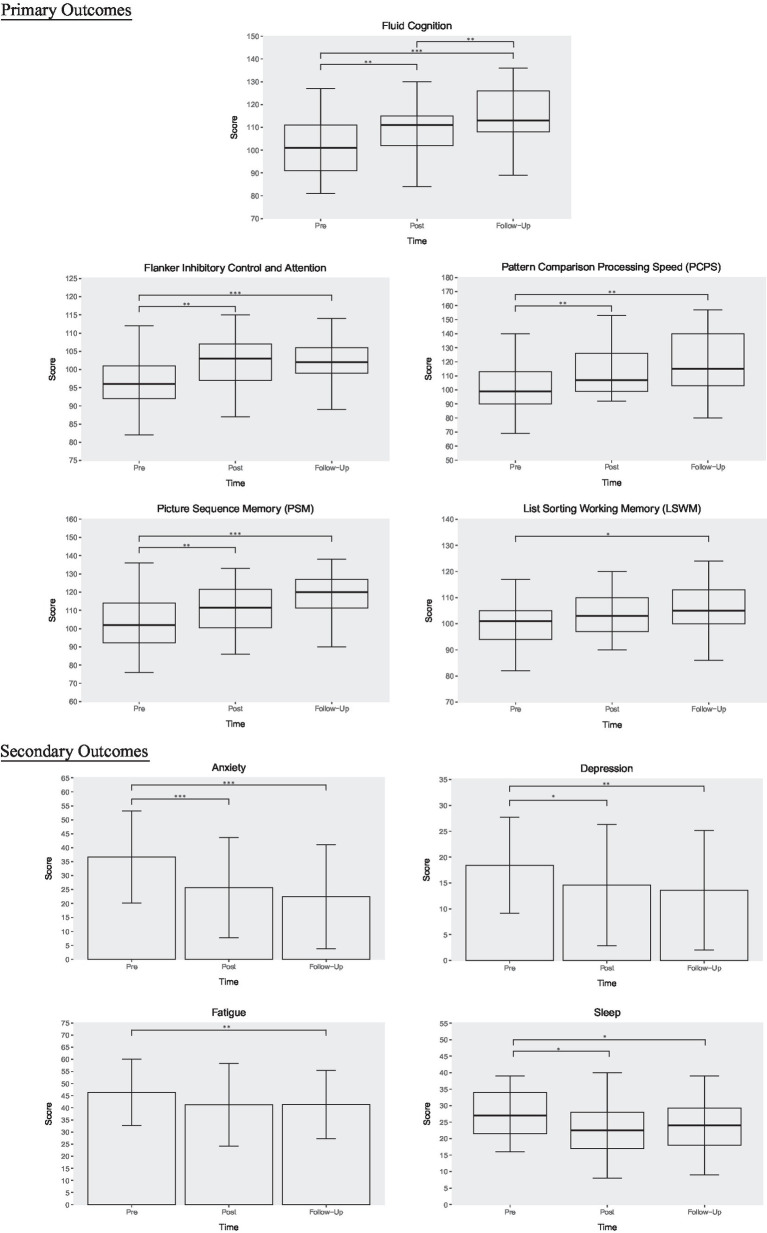
T-VNS intervention outcomes. This figure illustrates changes in cognition, anxiety, depression, fatigue, and sleep across three timepoints: pre-intervention, post-intervention, and 1-month follow-up. Boxplots are used for primarily nonparametric data, whereas column graphs depict parametric data. Error bars reflect standard error. ^*^p < 0.05, ^**^p < 0.01, ^***^p < 0.001.

Due to the absence of complete follow-up cognition data for six participants, Friedman’s test was run with *n* = 18 and revealed a main effect of time (*p* = 0.001) for fluid cognition (composite cognitive scores). *Post hoc* comparisons using Wilcoxon’s test with Bonferroni correction showed significant improvements from baseline to post-intervention (*p* = 0.003, r = 0.9) with further improvements at 1-month follow-up (pre vs. follow-up: *p* < 0.001, r = 0.98; post vs. follow-up: *p* = 0.003, r = 0.93) in composite fluid cognition. Given cognition was our primary outcome of interest, we further examined the cognitive subdomains. Significant gains were detected in Flanker Inhibitory Control and Attention from to pre to post (*p* = 0.009, r = 0.7) and from pre to follow-up (*p* < 0.001, r = 0.95). Moreover, there were significant increases from pre to post (*p* = 0.006, r = 0.86) and from pre to follow-up (*p* = 0.002, r = 0.94) in Pattern Comparison Processing Speed. Since Pattern Sequencing Memory (PSM) and List Sorting Working Memory (LSWM) contained the full dataset (*n* = 24) across all three timepoints and were normally distributed, they were analyzed with parametric tests. PSM scores significantly increased at post-intervention (*p* = 0.004, d = 0.55) and follow-up (*p* < 0.001, d = 0.82) when compared to baseline. However, LSWM only demonstrated improvements from baseline to follow-up (*p* = 0.029, d = 0.55). For Dimensional Change Card Sort test scores, a trending significance was found between pre-intervention and follow-up but did not survive multiple comparisons correction (*p* = 0.07). We additionally analyzed pre and post cognition scores available from all 24 patients and found similar results as the 18 patients but with slightly higher significance. For consistency and ease of visualization, the cognition graphs in Figure 3 reflect data from 18 patients, with the exception of PSM and LSWM (*n* = 24).

### 3.3 Secondary outcomes

Results from 24 participants revealed a main effect of time for anxiety [*F*(2, 46) = 14.46, *p* < 0.001], depression [*F*(2, 46) = 7.05, *p* = 0.002], sleep (*p* = 0.02), and fatigue [*F*(1.43, 32.86) = 4.13, *p* = 0.037]. *Post hoc* testing using Bonferroni correction revealed significant improvements from baseline to post-intervention and 1-month follow-up for anxiety (pre vs. post: *p* = 0.001, d = 0.619; pre vs. follow-up: *p* < 0.001, d = 0.803), depression (pre vs. post: *p* = 0.025, d = 0.351; pre vs. follow-up: *p* = 0.009, d = 0.443), and sleep (pre vs. post: *p* = 0.036, r = 0.6; pre vs. follow-up: *p* = 0.021, r = 0.66). Fatigue improved from baseline to 1 month follow-up only (*p* = 0.005, d = 0.335). No significant changes in smell were detected.

## 4 Discussion

This pilot study provides preliminary evidence that transcutaneous vagus nerve stimulation may offer a promising non-invasive approach to managing multiple symptoms in female patients with post-COVID-19 condition (Long COVID). We observed significant improvements across cognitive performance, mood (anxiety and depression), sleep, and fatigue following 10 days of t-VNS. Although pre-post changes in fatigue did not meet statistical significance, fatigue continued to decrease at 1-month follow-up, eventually reaching significance. Similarly, all outcomes except smell exhibited sustained gradual improvements even after cessation of treatment. Limited research has examined the retention of benefits from non-invasive VNS; thus, our findings provide initial evidence of potential long-term benefits warranting further controlled investigation. However, t-VNS did not lead to significant changes in olfactory performance.

The vagus nerve is a mixed nerve with approximately 80% afferent (sensory) and 20% efferent axonal projections. Stimulation of the auricular branch is thought to activate these afferent pathways, which then signal to the nucleus of the solitary tract in the brainstem to project to other central structures, leading to a cascade of neurophysiological changes that may potentially produce therapeutic effects (21). While the mechanism of action for the therapeutic benefits of VNS is still under investigation, transcutaneous VNS has been shown to affect the same neural pathway as invasive VNS (30). Potential mechanisms underlying VNS efficacy could include attenuation of systemic inflammation (17, 18, 24), an increase in cerebral blood flow (31, 32), normalization of autonomic function (33), and modulation of neurotransmitters (34, 35). Additionally, vagal neuromodulation also influences the hypothalamic–pituitary–adrenal (HPA) axis, which controls the body’s response to stress and regulates many processes, including mood and energy levels (17). Notably, conditions such as depression and anxiety, which can exacerbate inflammatory states leading to more fatigue, may be ameliorated through these pathways (36).

Cognitive enhancements with moderate to large effect sizes spanned multiple domains including attention, processing speed, episodic memory, working memory, and overall fluid cognition. Previous research has shown improvements in various aspects of cognition following non-invasive VNS in healthy adults, including cognitive flexibility (37), action control (38), post-error slowing (39), and different types of memory (40, 41). Furthermore, t-VNS mediated cognitive enhancements have also been reported in patients with neurological and neuropsychiatric disorders (42–45). Additionally, we observed clinically meaningful reductions in symptoms of anxiety and depression. Prior studies corroborate the antidepressant and anxiolytic effects of t-VNS (22, 46–48). Furthermore, t-VNS also alleviated sleep disturbances and fatigue severity, consistent with previous reports in insomnia patients (35, 49).

The current findings extend the potential multifaceted benefits of VNS to individuals with Long COVID. The improvements in our study are also partially supported by existing COVID-19 studies. Non-invasive vagus nerve stimulation has demonstrated success in attenuating symptoms in acute and chronic COVID-19 conditions. Using a similar t-VNS protocol with the Parasym device, Verbanck et al. (25) also reported improvements in depression, fatigue, cognition, and sleep from a pilot study of 20 Long COVID patients. However, cognition and sleep scores were not evaluated using validated scales and were grouped together as part of a composite personal score along with other symptoms, precluding the delineation of individual symptom attenuations. Our research builds upon these initial findings, offering a granular analysis with validated scales that distinctly evaluates cognitive and sleep scores. Moreover, Badran et al. (50) investigated the efficacy of t-VNS in 13 long-haulers using a double-blind randomized controlled trial (RCT). Though they did not find significant quantitative differences (underpowered), they reported a qualitative trend of t-VNS improving more than sham in mental fatigue. Qualitative differences in memory and attention have also been reported in acute, hospitalized COVID-19 patients (*n* = 21), when comparing t-VNS with sham (24). The lack of significant quantitative differences observed in these RCT studies may be attributable to several factors, including the heterogeneous nature of the patient population, the limited statistical power arising from small sample sizes, and the potential sex differences in treatment response. By focusing on an exclusively female cohort, our study aims to control for heterogeneity and provide detailed insights into the therapeutic responses of female long haulers to t-VNS. While our research cannot directly compare sex-dependent responses due to the absence of a male cohort, the therapeutic effects of t-VNS observed in women—who are disproportionately affected by Long COVID—may also extend to male patients. This targeted approach lays the groundwork for future comparative studies to explore potential sex differences in treatment responses.

As an initial pilot investigation, the lack of a control group limits definitive conclusions regarding the efficacy of t-VNS for Long COVID symptom management. However, the moderate to large effect sizes observed make significant placebo effects less likely. Additionally, robust randomized controlled trials of VNS have demonstrated significant benefits in various neurological and psychiatric conditions (51–53), which implies that the observed benefits in our study are less likely to be solely attributed to placebo effects. Though spontaneous recovery cannot be excluded given the lack of a control group, the chronicity of symptoms at enrollment (average 20.2 months post-infection) renders spontaneous recovery in a short 3-week period less likely. Moving forward, large, double blind, randomized, sham-controlled trials with extended follow-up should further evaluate t-VNS efficacy. Our promising pilot results provide preliminary evidence to justify additional rigorous evaluation and larger investments for such trials.

In conclusion, this pilot study suggests that transcutaneous vagal nerve stimulation may be a potential therapeutic approach for ameliorating a spectrum of persistent symptoms in females with Long COVID. Notably, we observed significant changes in cognition, mood, sleep, and fatigue, with potential long-term retention of benefits. However, due to the lack of a control group, we cannot exclude the possibility of other factors contributing to the observed improvements. To substantiate the efficacy of t-VNS and to address the limitations of our pilot study, subsequent research should include larger, double-blind, randomized, sham-controlled trials with extended follow-up periods. While our study did not encompass male participants, the robust responses in our female cohort hint at the potential universality of t-VNS benefits, laying the groundwork for future research to explore and validate these effects across sexes. Future investigations delving into the biological underpinnings of t-VNS’s therapeutic action can help pave the way for precision medicine approaches in Long COVID management.

## Data availability statement

The raw data supporting the conclusions of this article will be made available by the authors, without undue reservation.

## Ethics statement

The studies involving humans were approved by Institutional Review Board at Casa Colina Hospital and Centers for Healthcare. The studies were conducted in accordance with the local legislation and institutional requirements. The participants provided their written informed consent to participate in this study.

## Author contributions

ZZ: Conceptualization, Data curation, Formal analysis, Funding acquisition, Investigation, Methodology, Project administration, Resources, Supervision, Validation, Visualization, Writing – original draft, Writing – review & editing. NS: Data curation, Formal analysis, Visualization, Writing – review & editing. JW: Data curation, Investigation, Methodology, Writing – review & editing. ER: Project administration, Resources, Writing – review & editing.

## References

[1] Soriano JB Murthy S Marshall JC Relan P Diaz JV WHO Clinical Case Definition Working Group on Post-COVID-19 Condition. A clinical case definition of post-COVID-19 condition by a Delphi consensus. Lancet Infect Dis. (2022) 22:e102–7. doi: 10.1016/S1473-3099(21)00703-9, PMID: 34951953 PMC8691845

[2] Nittas V Gao M West EA Ballouz T Menges D Wulf Hanson S. Long COVID through a public health Lens: an umbrella review. Public Health Rev. (2022) 43:1604501. doi: 10.3389/phrs.2022.1604501, PMID: 35359614 PMC8963488

[3] Bai F Tomasoni D Falcinella C Barbanotti D Castoldi R Mulè G. Female gender is associated with long COVID syndrome: a prospective cohort study. Clin Microbiol Infect. (2021) 28:611.e9–611.e16. doi: 10.1016/j.cmi.2021.11.002, PMID: 34763058 PMC8575536

[4] Lechien JR Chiesa-Estomba CM De Siati DR Horoi M Le Bon SD Rodriguez A. Olfactory and gustatory dysfunctions as a clinical presentation of mild-to-moderate forms of the coronavirus disease (COVID-19): a multicenter European study. Eur Arch Otorrinolaringol. (2020) 277:2251–61. doi: 10.1007/s00405-020-05965-1, PMID: 32253535 PMC7134551

[5] Meng X Deng Y Dai Z Meng Z. COVID-19 and anosmia: a review based on up-to-date knowledge. Am J Otolaryngol. (2020) 41:102581. doi: 10.1016/j.amjoto.2020.102581, PMID: 32563019 PMC7265845

[6] Sudre CH Murray B Varsavsky T Graham MS Penfold RS Bowyer RC. Attributes and predictors of long COVID. Nat Med. (2021) 27:626–31. doi: 10.1038/s41591-021-01292-y, PMID: 33692530 PMC7611399

[7] Yong SJ. Long COVID or post-COVID-19 syndrome: putative pathophysiology, risk factors, and treatments. Infect Dis. (2021) 53:737–54. doi: 10.1080/23744235.2021.1924397, PMID: 34024217 PMC8146298

[8] Peluso MJ Lu S Tang AF Durstenfeld MS Ho H-E Goldberg SA. Markers of immune activation and inflammation in individuals with Postacute sequelae of severe acute respiratory syndrome coronavirus 2 infection. J Infect Dis. (2021) 224:1839–48. doi: 10.1093/infdis/jiab490, PMID: 34677601 PMC8643408

[9] Rubtsov AV Rubtsova K Kappler JW Marrack P. Genetic and hormonal factors in female-biased autoimmunity. Autoimmun Rev. (2010) 9:494–8. doi: 10.1016/j.autrev.2010.02.008, PMID: 20144912 PMC3171140

[10] Taneja V. Sex Hormones Determine Immune Response. Front Immunol. (2018) 9:1931. doi: 10.3389/fimmu.2018.01931, PMID: 30210492 PMC6119719

[11] Graham EL Clark JR Orban ZS Lim PH Szymanski AL Taylor C. Persistent neurologic symptoms and cognitive dysfunction in non-hospitalized Covid-19 long haulers. Ann Clin Transl Neurol. (2021) 8:5:1073–85. doi: 10.1002/acn3.51350, PMID: 33755344 PMC8108421

[12] Douaud G Lee S Alfaro-Almagro F Arthofer C Wang C McCarthy P. SARS-CoV-2 is associated with changes in brain structure in UK biobank. Nature. (2022) 604:697–707. doi: 10.1038/s41586-022-04569-5, PMID: 35255491 PMC9046077

[13] Thomasson M Voruz P Cionca A Jacot de Alcântara I Nuber-Champier A Allali G. Markers of limbic system damage following SARS-CoV-2 infection. Brain Commun. (2023) 5:fcad177. doi: 10.1093/braincomms/fcad177, PMID: 37415776 PMC10320753

[14] Vilarello BJ Jacobson PT Tervo JP Waring NA Gudis DA Goldberg TE. Olfaction and neurocognition after COVID-19: a scoping review. Front Neurosci. (2023) 17:1198267. doi: 10.3389/fnins.2023.1198267 37457004 PMC10339825

[15] Rahman A Jackson H Hristov H Isaacson RS Saif N Shetty T. Sex and gender driven modifiers of Alzheimer’s: the role for estrogenic control across age, race, medical, and lifestyle risks. Front Aging Neurosci. (2019) 11:315. doi: 10.3389/fnagi.2019.00315, PMID: 31803046 PMC6872493

[16] Zou Y Lu D Liu L Zhang H Zhou Y. Olfactory dysfunction in Alzheimer’s disease. Neuropsychiatr Dis Treat. (2016) 12:869–75. doi: 10.2147/NDT.S104886, PMID: 27143888 PMC4841431

[17] Breit S Kupferberg A Rogler G Hasler G. Vagus nerve as modulator of the brain-gut Axis in psychiatric and inflammatory disorders. Front Psych. (2018) 9:44. doi: 10.3389/fpsyt.2018.00044, PMID: 29593576 PMC5859128

[18] Bonaz B Sinniger V Pellissier S. Anti-inflammatory properties of the vagus nerve: potential therapeutic implications of vagus nerve stimulation. J Physiol. (2016) 594:5781–90. doi: 10.1113/JP271539, PMID: 27059884 PMC5063949

[19] Boon P Moors I De Herdt V Vonck K. Vagus nerve stimulation and cognition. Seizure. (2006) 15:259–63. doi: 10.1016/j.seizure.2006.02.014 16651013

[20] Engineer ND Kimberley TJ Prudente CN Dawson J Tarver WB Hays SA. Targeted Vagus nerve stimulation for rehabilitation after stroke. Front Neurosci. (2019) 13:280. doi: 10.3389/fnins.2019.00280, PMID: 30983963 PMC6449801

[21] Howland RH. Vagus nerve stimulation. Curr Behav Neurosci Rep. (2014) 1:64–73. doi: 10.1007/s40473-014-0010-5 24834378 PMC4017164

[22] Kong J Fang J Park J Li S Rong P. Treating depression with transcutaneous auricular Vagus nerve stimulation: state of the art and future perspectives. Front Psych. (2018) 9:20. doi: 10.3389/fpsyt.2018.00020, PMID: 29459836 PMC5807379

[23] Tornero C Pastor E Garzando M Orduña J Forner MJ Bocigas I. Non-invasive Vagus Nerve Stimulation for COVID-19: Results From a Randomized Controlled Trial (SAVIOR I). Front Neurol. (2022) 13:864. doi: 10.3389/fneur.2022.820864 PMC9028764 35463130

[24] Uehara L Corrêa JCF Ritti R Leite P de Faria DRG Pacheco-Barrios K. Transcutaneous auricular vagus nerve stimulation effects on inflammatory markers and clinical evolution of patients with COVID-19: a pilot randomized clinical trial. Expert Rev Med Devices. (2022) 19:915–20. doi: 10.1080/17434440.2022.2154147, PMID: 36540947

[25] Verbanck C. (2021). Transcutaneous Auricular Vagus Nerve Stimulation (tVNS) can Reverse the Manifestations of the Long-COVID Syndrome: A Pilot Study. Available at: https://www.semanticscholar.org/paper/Transcutaneous-Auricular-Vagus-Nerve-Stimulation-of-Verbanck-Corazza/8a39f3f621d92576369d988a59bf617e3935cf36

[26] Yokota H Edama M Hirabayashi R Sekine C Otsuru N Saito K. Effects of stimulus frequency, intensity, and sex on the autonomic response to transcutaneous Vagus nerve stimulation. Brain Sci. (2022) 12:1038. doi: 10.3390/brainsci12081038, PMID: 36009101 PMC9405815

[27] Tsampasian V Elghazaly H Chattopadhyay R Debski M Naing TKP Garg P. Risk factors associated with post−COVID-19 condition: a systematic review and Meta-analysis. JAMA Intern Med. (2023) 183:566–80. doi: 10.1001/jamainternmed.2023.0750, PMID: 36951832 PMC10037203

[28] Troyanskaya O Cantor M Sherlock G Brown P Hastie T Tibshirani R. Missing value estimation methods for DNA microarrays. Bioinformatics. (2001) 17:520–5. doi: 10.1093/bioinformatics/17.6.520 11395428

[29] Huang J Keung JW Sarro F Li Y-F Yu YT Chan WK. Cross-validation based K nearest neighbor imputation for software quality datasets: an empirical study. J Syst Softw. (2017) 132:226–52. doi: 10.1016/j.jss.2017.07.012

[30] Yap JYY Keatch C Lambert E Woods W Stoddart PR Kameneva T. Critical review of transcutaneous Vagus nerve stimulation: challenges for translation to clinical practice. Front Neurosci. (2020) 14:284. doi: 10.3389/fnins.2020.00284 32410932 PMC7199464

[31] Fang J Egorova N Rong P Liu J Hong Y Fan Y. Early cortical biomarkers of longitudinal transcutaneous vagus nerve stimulation treatment success in depression. NeuroImage Clin. (2017) 14:105–11. doi: 10.1016/j.nicl.2016.12.016, PMID: 28180068 PMC5279909

[32] Vonck K De Herdt V Bosman T Dedeurwaerdere S Van Laere K Boon P. Thalamic and limbic involvement in the mechanism of action of vagus nerve stimulation, a SPECT study. Seizure. (2008) 17:699–706. doi: 10.1016/j.seizure.2008.05.001, PMID: 18556220

[33] Clancy JA Mary DA Witte KK Greenwood JP Deuchars SA Deuchars J. Non-invasive Vagus nerve stimulation in healthy humans reduces sympathetic nerve activity. Brain Stimul. (2014) 7:871–7. doi: 10.1016/j.brs.2014.07.031, PMID: 25164906

[34] Carreno FR Frazer A. Vagal nerve stimulation for treatment-resistant depression. Neurotherapeutics. (2017) 14:716–27. doi: 10.1007/s13311-017-0537-8, PMID: 28585221 PMC5509631

[35] Jiao Y Guo X Luo M Li S Liu A Zhao Y. Effect of transcutaneous Vagus nerve stimulation at auricular concha for insomnia: a randomized clinical trial. Evid Based Complement Alternat Med. (2020) 2020:e6049891:1–7. doi: 10.1155/2020/6049891, PMID: 32831871 PMC7429019

[36] Lee C-H Giuliani F. The role of inflammation in depression and fatigue. Front Immunol. (2019) 10:1696. doi: 10.3389/fimmu.2019.01696, PMID: 31379879 PMC6658985

[37] Borges U Knops L Laborde S Klatt S Raab M. Transcutaneous Vagus nerve stimulation May enhance only specific aspects of the Core executive functions. A randomized crossover trial. Front Neurosci. (2020) 14:523. doi: 10.3389/fnins.2020.00523, PMID: 32523510 PMC7262369

[38] Jongkees BJ Immink MA Finisguerra A Colzato LS. Transcutaneous Vagus nerve stimulation (tVNS) enhances response selection during sequential action. Front Psychol. (2018) 9:1159. doi: 10.3389/fpsyg.2018.01159, PMID: 30034357 PMC6043681

[39] Sellaro R van Leusden JWR Tona K-D Verkuil B Nieuwenhuis S Colzato LS. Transcutaneous Vagus nerve stimulation enhances post-error slowing. J Cogn Neurosci. (2015) 27:2126–32. doi: 10.1162/jocn_a_00851, PMID: 26226074

[40] Jacobs HIL Riphagen JM Razat CM Wiese S Sack AT. Transcutaneous vagus nerve stimulation boosts associative memory in older individuals. Neurobiol Aging. (2015) 36:1860–7. doi: 10.1016/j.neurobiolaging.2015.02.023, PMID: 25805212

[41] Jandackova V Vasendova V Jackowska M Koenig J. The effect of long-term non-invasive vagus nerve stimulation on cognitive performance: results from a randomized placebo controlled trial. Brain Stimul. (2023) 16:6. doi: 10.1016/j.brs.2023.03.028

[42] Aniwattanapong D List JJ Ramakrishnan N Bhatti GS Jorge R. Effect of Vagus nerve stimulation on attention and working memory in neuropsychiatric disorders: a systematic review. Neuromodulation. (2022) 25:343–55. doi: 10.1016/j.neurom.2021.11.009, PMID: 35088719

[43] Broncel A Bocian R Kłos-Wojtczak P Kulbat-Warycha K Konopacki J. Vagal nerve stimulation as a promising tool in the improvement of cognitive disorders. Brain Res Bull. (2020) 155:37–47. doi: 10.1016/j.brainresbull.2019.11.011, PMID: 31790720

[44] Giraudier M Ventura-Bort C Weymar M. Transcutaneous Vagus nerve stimulation (tVNS) improves high-confidence recognition memory but not emotional word processing. Front Psychol. (2020) 11:1276. doi: 10.3389/fpsyg.2020.01276, PMID: 32733306 PMC7363946

[45] Vargas-Caballero M Warming H Walker R Holmes C Cruickshank G Patel B. Vagus nerve stimulation as a potential therapy in early Alzheimer’s disease: a review. Front Hum Neurosci. (2022) 16:866434. doi: 10.3389/fnhum.2022.866434, PMID: 35572001 PMC9098960

[46] George MS Ward HE Ninan PT Pollack M Nahas Z Anderson B. A pilot study of vagus nerve stimulation (VNS) for treatment-resistant anxiety disorders. Brain Stimul. (2008) 1:112–21. doi: 10.1016/j.brs.2008.02.001, PMID: 20633378

[47] Grolaux PJD. Transcutaneous Vagus nerve stimulation in private healthcare center: a small-scale investigation targeting anxiety, irritable bowel syndrome and chronic pain. J Neurol Neuromed. (2019) 4:7–22. doi: 10.29245/2572.942X/2019/5.1251

[48] Hein E Nowak M Kiess O Biermann T Bayerlein K Kornhuber J. Auricular transcutaneous electrical nerve stimulation in depressed patients: a randomized controlled pilot study. J Neural Transm. (2013) 120:821–7. doi: 10.1007/s00702-012-0908-6, PMID: 23117749

[49] Wu D Wang J Yuan Y. Effects of transcranial direct current stimulation on naming and cortical excitability in stroke patients with aphasia. Neurosci Lett. (2015) 589:115–20. doi: 10.1016/j.neulet.2015.01.045, PMID: 25603474

[50] Badran BW Huffman SM Dancy M Austelle CW Bikson M Kautz SA. A pilot randomized controlled trial of supervised, at-home, self-administered transcutaneous auricular vagus nerve stimulation (taVNS) to manage long COVID symptoms. Bioelectron Med. (2022) 8:13. doi: 10.1186/s42234-022-00094-y, PMID: 36002874 PMC9402278

[51] Cimpianu C-L Strube W Falkai P Palm U Hasan A. Vagus nerve stimulation in psychiatry: a systematic review of the available evidence. J Neural Transm. (2017) 124:145–58. doi: 10.1007/s00702-016-1642-2, PMID: 27848034

[52] Vonck K Raedt R Naulaerts J De Vogelaere F Thiery E Van Roost D. Vagus nerve stimulation…25 years later! What do we know about the effects on cognition? Neurosci Biobehav Rev. (2014) 45:63–71. doi: 10.1016/j.neubiorev.2014.05.005, PMID: 24858008

[53] Wang L Zhang J Guo C He J Zhang S Wang Y. The efficacy and safety of transcutaneous auricular vagus nerve stimulation in patients with mild cognitive impairment: a double blinded randomized clinical trial. Brain Stimul. (2022) 15:1405–14. doi: 10.1016/j.brs.2022.09.003 36150665

