# Supplementary material for: Transcutaneous vagus nerve stimulation improves Long COVID symptoms in a female cohort: a pilot study
Source: Front Neurol. 2024 May 2;15:1393371. doi: 10.3389/fneur.2024.1393371 (PMC11097097; doi:10.3389/fneur.2024.1393371)
Supplement: Supplementary file 1 [file Table_1.DOCX]

**Supplementary Figure 1. Medication categories and their prevalence based on 24 participants**

**
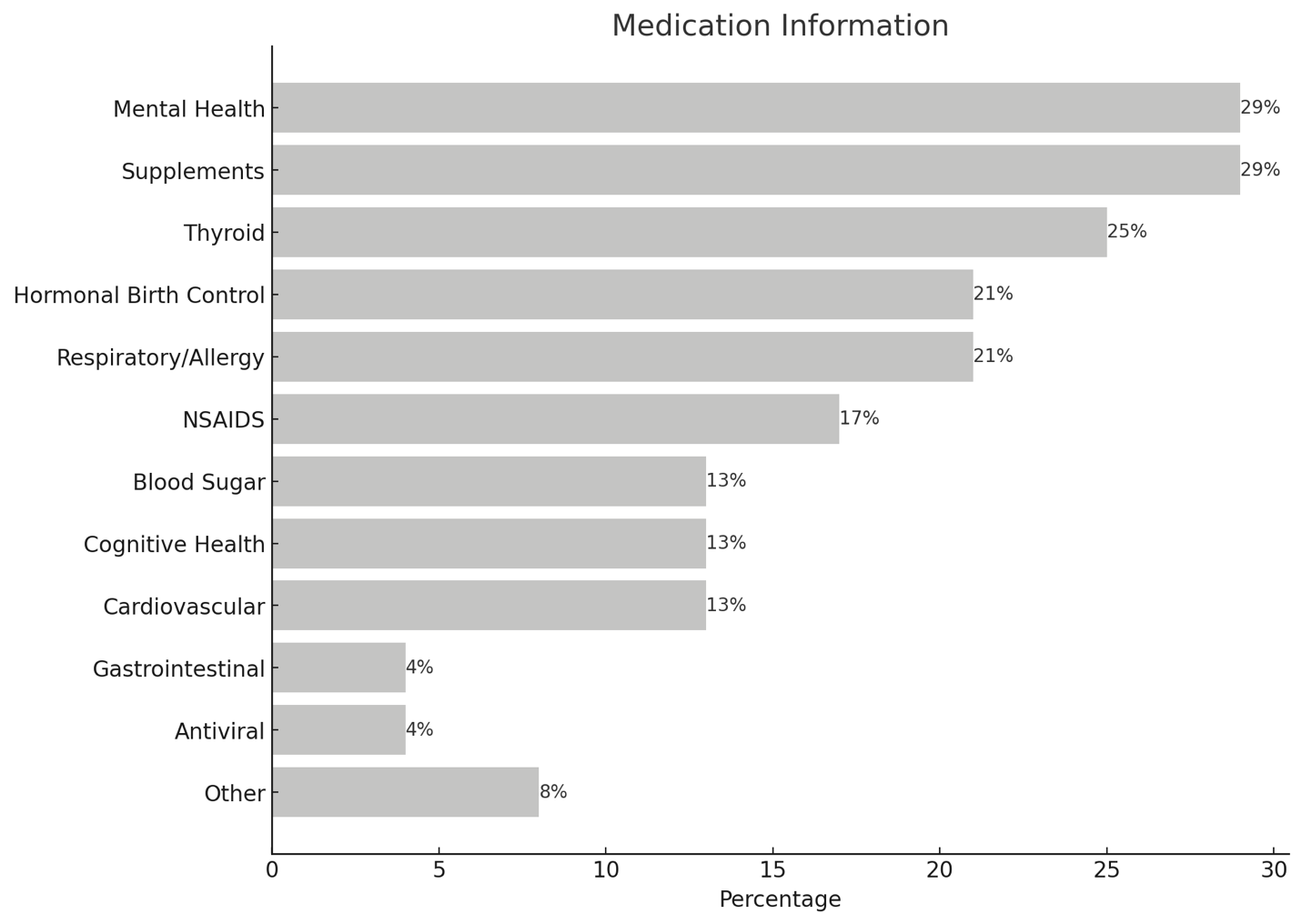
**

**Supplementary Table 1. Medication Categories and Names**

| **Category** | **Medication** |
| --- | --- |
| Mental Health | Fluoxetine, Zoloft, Fluvoxamine, Lexapro, Xanax, Effexor, Amitriptyline, Bupropion |
| Supplements | Vitamins, Minerals, Pre/probiotics, Ubiquinol, Quercetin, DHA-EPA, Alpha lipoic acid |
| Thyroid | Armour Thyroid, Levothyroxine, Liothyronine, Methimazole |
| Hormonal Birth Control | Combined oral contraceptive pills, Progesterone, Nexplanon |
| Respiratory/Allergy | Albuterol, Alvesco, Diphenhydramine, Cetrizine, Montelukast, Flotucasone, Zyrtec |
| NSAIDS | Aspirin, Ibuprofen, Naproxen, Meloxicam |
| Blood Sugar | Metformin, Glipizide, Insulin Aspart |
| Cognitive Health | Adderall |
| Cardiovascular | Metoprolol, Statins, Nexlizet |
| Gastrointenstinal | Pepcid |
| Antiviral | Famciclovir |
| Other | Low-dose Naltrexone, Oxybutynin Chloride |
